# Crystal structure of *Epiphyas postvittana* pheromone binding protein 3

**DOI:** 10.1038/s41598-020-73294-8

**Published:** 2020-10-01

**Authors:** Cyril Hamiaux, Colm Carraher, Christer Löfstedt, Jacob A. Corcoran

**Affiliations:** 1 The New Zealand Institute for Plant and Food Research Limited, Auckland, New Zealand; 2 Department of Biology, Lund University, Lund, Sweden; 3 Biological Control of Insects Research Laboratory, USDA - Agricultural Research Service, Columbia, MO USA

**Keywords:** X-ray crystallography, Entomology, Molecular modelling

## Abstract

The insect olfactory system operates as a well-choreographed ensemble of molecules which functions to selectively translate volatile chemical messages present in the environment into neuronal impulses that guide insect behaviour. Of these molecules, binding proteins are believed to transport hydrophobic odorant molecules across the aqueous lymph present in antennal sensilla to receptors present in olfactory sensory neurons. Though the exact mechanism through which these proteins operate is still under investigation, these carriers clearly play a critical role in determining what an insect can smell. Binding proteins that transport important sex pheromones are colloquially named pheromone binding proteins (PBPs). Here, we have produced a functional recombinant PBP from the horticultural pest, *Epiphyas postvittana* (EposPBP3), and experimentally solved its apo-structure through X-ray crystallography to a resolution of 2.60 Å. Structural comparisons with related lepidopteran PBPs further allowed us to propose models for the binding of pheromone components to EposPBP3. The data presented here represent the first structure of an olfactory-related protein from the tortricid family of moths, whose members cause billions of dollars in losses to agricultural producers each year. Knowledge of the structure of these important proteins will allow for subsequent studies in which novel, olfactory molecule-specific insecticides can be developed.

## Introduction

Insects rely on their sense of smell for detecting food, mates and oviposition sites, among other things. Although the exact mechanism through which this system operates is not fully known, there are several proteins involved. Insect Pheromone Binding Proteins (PBPs) and Odorant Binding Proteins (OBPs) are found at very high concentrations (up to 10 μM) in the sensillum lymph of insect antennae^1^. These proteins form a large multigene family of small soluble proteins (~ 15 kDa) believed to function as carrier molecules to transport hydrophobic compounds across the sensillum lymph to the membrane-bound odorant receptors present in olfactory sensory neurons (OSNs). There is considerable functional and structural data available on PBPs/OBPs from various insect species^2–4^ yet the exact role, specificity and mechanism of ligand binding and release remains controversial^4^.

PBP/OBPs are α-helical proteins of ~ 150 amino acids. The first crystal structure for a PBP/OPB was of PBP1 from the silk moth *Bombyx mori* (BmorPBP1) bound to the sex pheromone (bombykol, (*E*,*Z*)-10,12-hexadecadien-1-ol), which established the classical PBP/OBP fold consisting of six α-helices stabilized by three disulphide bridges^5^. The arrangement of the helices builds an internal, mostly hydrophobic, cavity that accommodates and fully encloses the bound pheromone^5^. Prior biochemical characterization of BmorPBP1 indicated that the protein undergoes conformational changes with a reduction in pH^6^. This was confirmed when the NMR structure of BmorPBP1 at pH 4.5 showed that the C-terminal portion of the protein (that adopted an elongated conformation in the ligand-bound structure) became folded as an additional α-helix that occupied the pheromone binding cavity of the protein, while the N-terminal part of the protein (that formed a cavity-closing α-helix in the ligand-bound form) adopted a fully elongated conformation^7^. The acidic and basic forms of BmorPBP1 are referred to as forms A and B, respectively. Based on these results, a pH-driven mechanism for ligand binding and release of BmorPBP1 was hypothesized, where the ligand binds at neutral pH within the sensillum lymph and is released near the membrane surface (where the pH is presumably more acidic) by the folding of the C-terminal helix inside the binding cavity^7–10^. A similar pH-dependent conformational change has been observed for two other lepidopteran PBPs, *Amyelois transitella* PBP1^11–13^ and *Antheraea polyphemus* PBP1^14^, suggesting that the pH-driven proposed mechanism for ligand binding and release is likely to be broadly conserved amongst lepidopteran PBPs. In the case of BmorPBP1, however, a crystal structure of the apo protein at pH 7.5 was later observed to adopt a conformation very close to form A, suggesting that the conformational change may also be ligand regulated^8^. More generally, in light of the high diversity in insect physiology, it is likely that other mechanisms for ligand binding and release exist for PBPs/OBPs^2^.

Adding to the existing knowledge base of these important proteins will help to further understand the complex interactions that occur in the insect olfactory system. It has been suggested that OBPs could be useful in designing novel pest management strategies^4^, however these applications should be approached cautiously, as it has been proposed that new control strategies targeting olfactory proteins with low specificity may lead to many of the issues associated with broad-spectrum insecticides^15^. In addition, it has been shown recently that binding proteins can be exploited as the foundational components of next-generation biosensors^16–18^. A better understanding of how the binding proteins recognize and interact with their ligands is critical for their usage in these applications.

The light brown apple moth, *Epiphyas postvittana*, is a horticultural pest that originated in Australia, which has now established itself in New Zealand, several European countries, Hawaii and California^19,20^. The sex pheromone of *E. postvittana* consists of a blend of two main components, (*E*)-11-tetradecenyl acetate (E11-14:OAc) and (*E*,*E*)-9,11-tetradecadienyl acetate (E9,E11-14:OAc), and two minor components, (*E*)-11-tetradecen-1-ol (E11-14:OH) and (*E*)-11-hexadecenyl acetate (E11-16:OAc), in a ratio of 200:5:2:1, respectively^21,22^. The peripheral olfactory repertoire of *E. postvittana* is now well characterized. Three PBPs (EposPBP1, PBP2 and PBP3) were originally identified from *E. postvittana* antennae through expressed-sequence tag and proteomic analyses^23,24^. Subsequent transcriptomic analyses of adult antennae^25^ identified an additional 31 OBPs in *E. postvittana*, and phylogenetic analyses confirmed that EposPBPs 1, 2 and 3 were the only OBPs closely related to the well-defined 3-disulphide bond containing lepidopteran PBP clade. EposPBP1 has been shown to bind the major component of the sex pheromone in a binding assay with radiolabelled ligands^23^. EposPBP1 and PBP3 were found to be expressed at higher levels in male vs. female antennae, while EposPBP2 has higher expression levels in female antennae^24^. There is no structural information available for any *E. postvittana* PBP or OBP to date. In the present report, we have recombinantly expressed EposPBP3 in insect cells, showed that this protein was functional and solved its structure to 2.60 Å resolution.

## Results

### Recombinant protein production

Initial attempts to express and purify EposPBP3 carrying a cleavable N-terminal His-tag in *E. coli* were successful, however no crystals were obtained with that construct. In subsequent attempts we designed a new construct carrying a C-terminal His-tag but it failed to express in *E. coli* and only showed extremely low initial expression levels in Sf9 cells using DH10Bac. In an attempt to improve expression levels we compared DH10Bac, MultiBac and EmbacY derived viruses, however, the levels in SF9 cells were similarly low. We then tested these three baculovirus strains in High Five cells and were able to successfully produce high levels of recombinant EposPBP3 using the EmBacY system. The protein was secreted into the media and purified by affinity chromatography, followed by size exclusion chromatography where it eluted at the correct size for a monomer.

### Functional testing of EposPBP3

**Figure 1:**
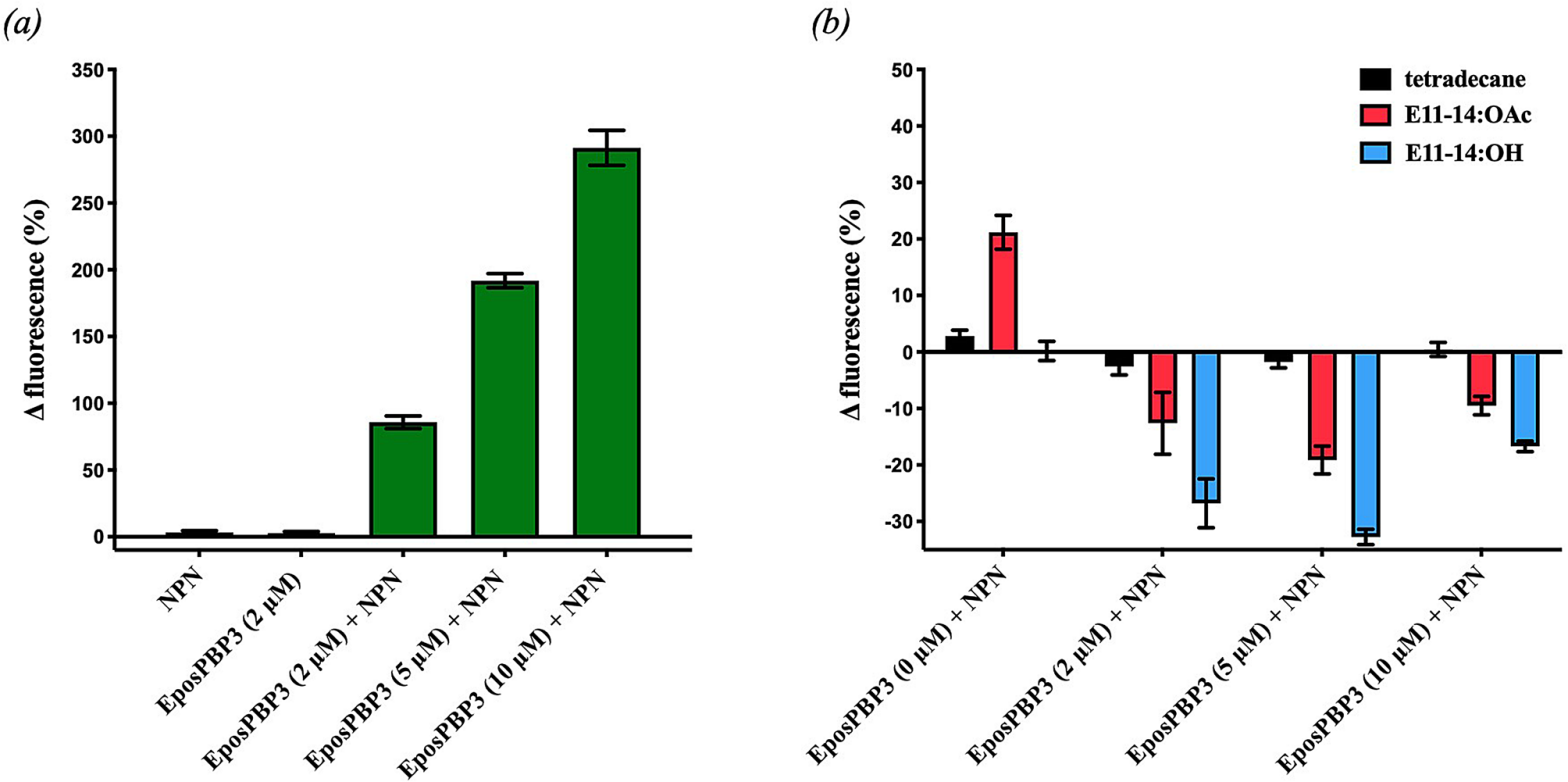
Functional testing of recombinant EposPBP3 using the fluorescent indicator, N-Phenyl-1-napthylamine (NPN). (**a**) Mean (± SEM) change in fluorescence upon addition of NPN (10 μM) or EposPBP3 (2 μM) to assay buffer, or upon addition of indicated concentrations of EposPBP3 to buffer containing 10 μM NPN. (**b**) Mean (± SEM) change in fluorescence upon addition of pheromone compounds or control (10 μM) to assay buffer containing indicated concentrations of EposPBP3 and NPN (10 μM).

Initial fluorescence measurements found very little change in fluorescence when the fluorescent probe N-phenyl-1-napthylamine (NPN) or EposPBP3 was added to the assay buffer solution alone and excited at 337 nm, however, when EposPBP3 was added to buffer containing 10 μM NPN, a dose-dependent increase in fluorescent emission was observed (Fig. 1). We then performed competitive binding experiments by comparing changes in fluorescence upon addition of pheromone compounds to wells containing 10 μM NPN and various concentrations of EposPBP3. We found that fluorescence emissions decreased substantially upon addition of E11-14:OAc or E11-14:OH, but not tetradecane, to wells containing 2 μM and 5 μM EposPBP3, suggesting these compounds were able to disrupt the NPN/EposPBP3 interaction due to their intrinsic affinities for the binding site of EposPBP3. When the test compounds were added to wells containing 10 μM EposPBP3 the fluorescent signals decreased, albeit less than they did at lower PBP concentrations, which is likely indicative of an impaired solubility capacity of the system at such high protein concentrations. Interestingly, in control experiments, in which the compounds were added to wells containing NPN only (0 μM EposPBP3), an increase in fluorescence was observed with E11-14:OAc, suggesting this compound interacted with the fluorescent probe and caused its emission spectra to change, similar to that of EposPBP3 binding (Fig. 1). For this reason, due to the obvious complications imposed on competitive binding assays using NPN and E11-14:OAc, no further binding experiments (e.g., affinity, kinetic determinations) with these compounds or the other pheromone blend components (E9,E11-14:OAc and E11-16:OAc) were performed.

### Crystal structure of EposPBP3

**Figure 2:**
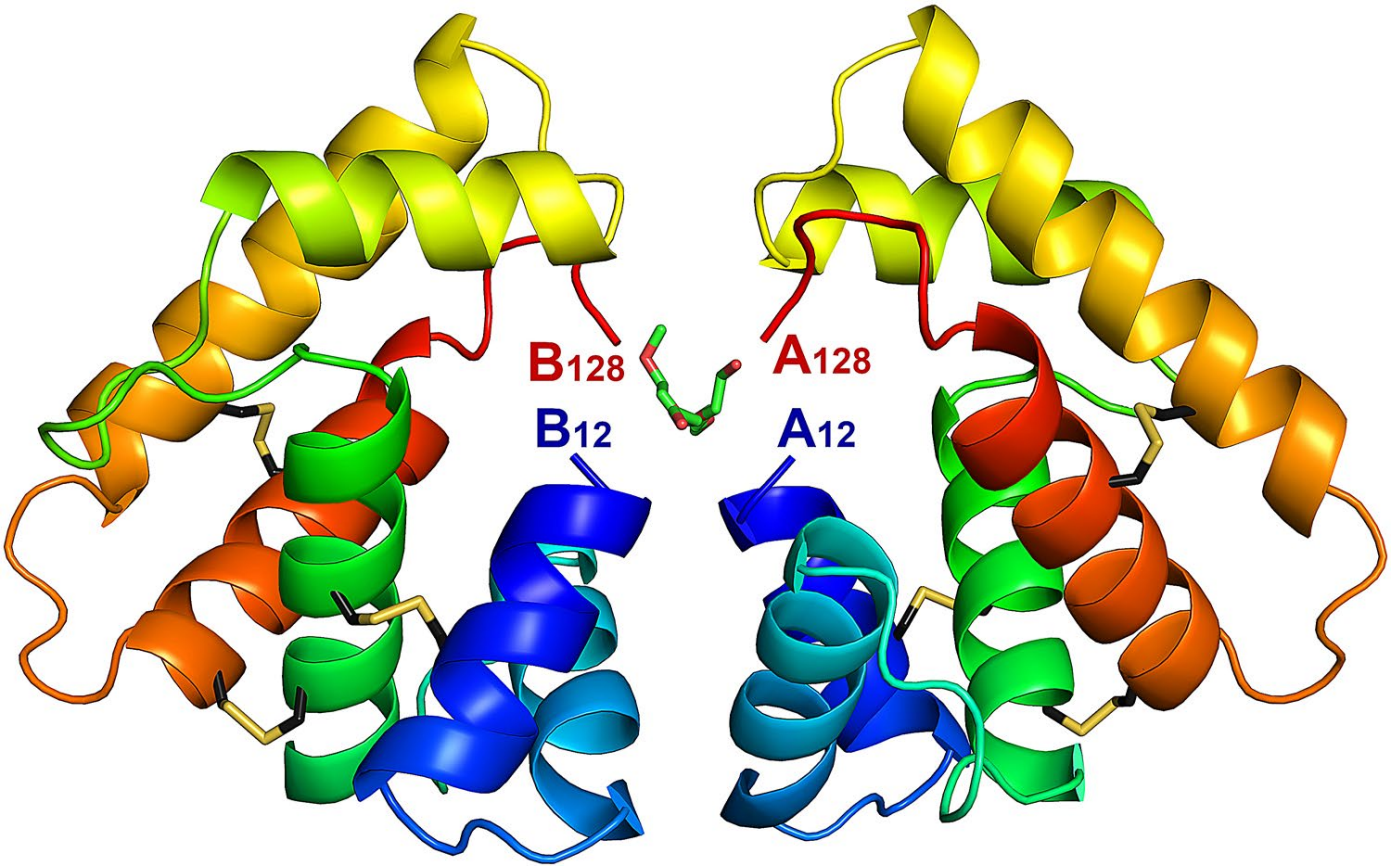
Crystal structure of EposPBP3 with two molecules in the asymmetric unit. Each molecule is shown in ribbon mode and is rainbow coloured from blue (N-terminal) to red (C-terminal). The residue numbers of the N- and C-termini of each protomer (**A**,**B**) are indicated. The disulphide bridges are drawn in stick mode, with carbon atoms in black and sulphur atoms in yellow. PEG molecules are drawn in stick mode, with carbon atoms in green and oxygen atoms in red. Image drawn using PyMOL Molecular Graphics System, Version 2.0 (https://pymol.org/2/).

Two crystal forms of EposPBP3 were obtained in two conditions of the Structure Screen 1 + 2 (Molecular Dimensions) after 11 months of incubation. Crystals from the SS2-46 condition (10% PEG 1000, 10% PEG 8000) only showed diffraction to ~ 8.00 Å and data were not collected. Crystals from the SS2-22 condition (0.1 M MES, pH 6.5, 12% PEG 20,000), however, showed good diffraction patterns and a full dataset was collected to a resolution of 2.60 Å. Crystals belonged to the P4_3_ space group, with two molecules in the asymmetric unit. The structure was solved by molecular replacement using the structure of BmorPBP1^5^ as a search model (PDB entry 1DQE). As shown in Fig. 2, the overall fold of EposPBP3 is conserved with other insect PBPs/OBPs, consisting of six α-helices stabilized by three disulphide bridges (Cys19–Cys54, Cys50–Cys108, and Cys97–Cys117). In contrast to other PBP/OBP structures available in the PDB, both the N-terminus (residues 1–11) and the C-terminus (residues 129–159, which includes the TEV cleavage site and the C-terminal His-Tag) of EposPBP3 appear to be disordered and therefore were not modelled. This may be a consequence of the packing arrangement of the two molecules in the asymmetric unit that brings the N- and C-termini of both molecules in close proximity, where folded N- or C-terminal parts of each monomer would result in steric clashes that are incompatible with the dimer interface observed in the crystals (Fig. 2). Towards the end of the refinement, two residual elongated stretches of electron density that could not be interpreted as protein atoms remained at the dimer interface (between the N- and C-termini of both molecules). These were modelled as PEG fragments originating from the crystallisation solution (Fig. 2 and Supplementary Fig. S1).

**Figure 3:**
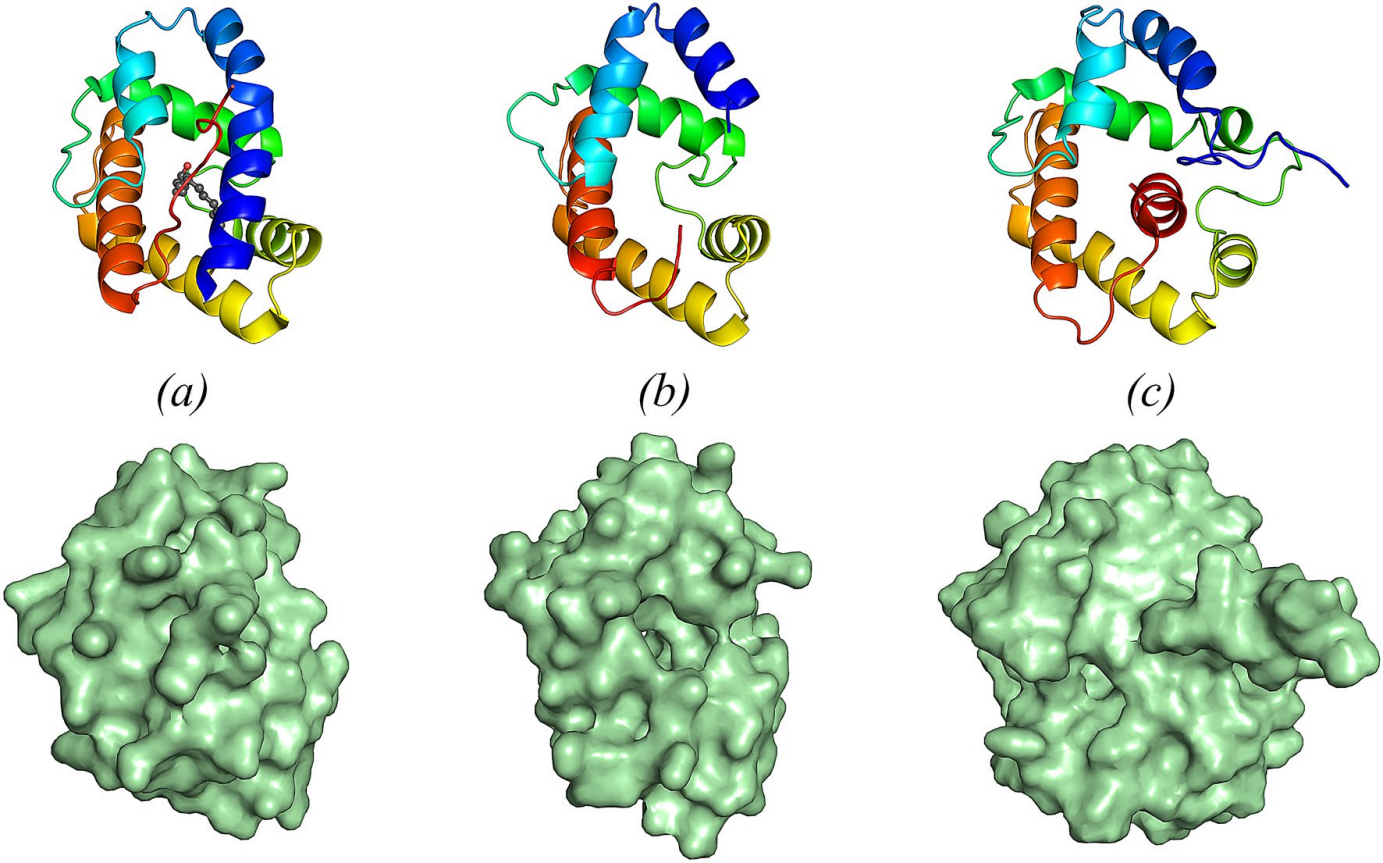
Structure (top) and surface representation (bottom) of (**a**) the ligand-bound form (form B) of BmorPBP1 (1–137), bound to the natural pheromone bombykol (PDB 1DQE), (**b**) EposPBP3 (12–128) and (**c**) BmorPBP1 (1–142) in solution at pH 4.5 (form A) (PDB 1GM0). All structures are shown in ribbon mode and coloured from blue (N-terminus) to red (C-terminus). Bombykol is shown in sphere mode in (**a**), with carbon and oxygen atoms coloured dark grey and red, respectively. Image drawn using PyMOL Molecular Graphics System, Version 2.0 (https://pymol.org/2/).

A DALI search identified BmorPBP1 in form B (bound to the non-natural ligand iodohexadecane, PDB 2P71) as the closest structural homologue of EposPBP3 (Z score = 15.6, rmsd = 1.80 Å for Cα atoms of 112 aligned residues). As mentioned previously, BmorPBP1 has been observed in two different conformations: a ligand-bound form at neutral and basic pH, characterized by a fully folded N-terminal helix and an elongated C-terminal extension (form B)^5,26^, and a form at acidic pH, where the C-terminal part of the protein folds into an additional α-helix that occupies the binding cavity, while the N-terminus adopts an elongated conformation (form A). In contrast to form A and form B of BmorPBP1, however, EposPBP3 has both its N- and C-termini disordered in the crystal. The structure obtained for EposPBP3 is therefore best described as an intermediate form between form A (N-terminal extended, and C-terminal helix folded) and form B (N-terminal helix folded, C-terminal extended) (Fig. 3). Consequently, and in contrast to BmorPBP1 structures, EposPBP3 harbours a widely open internal cavity (Fig. 3).

**Figure 4:**
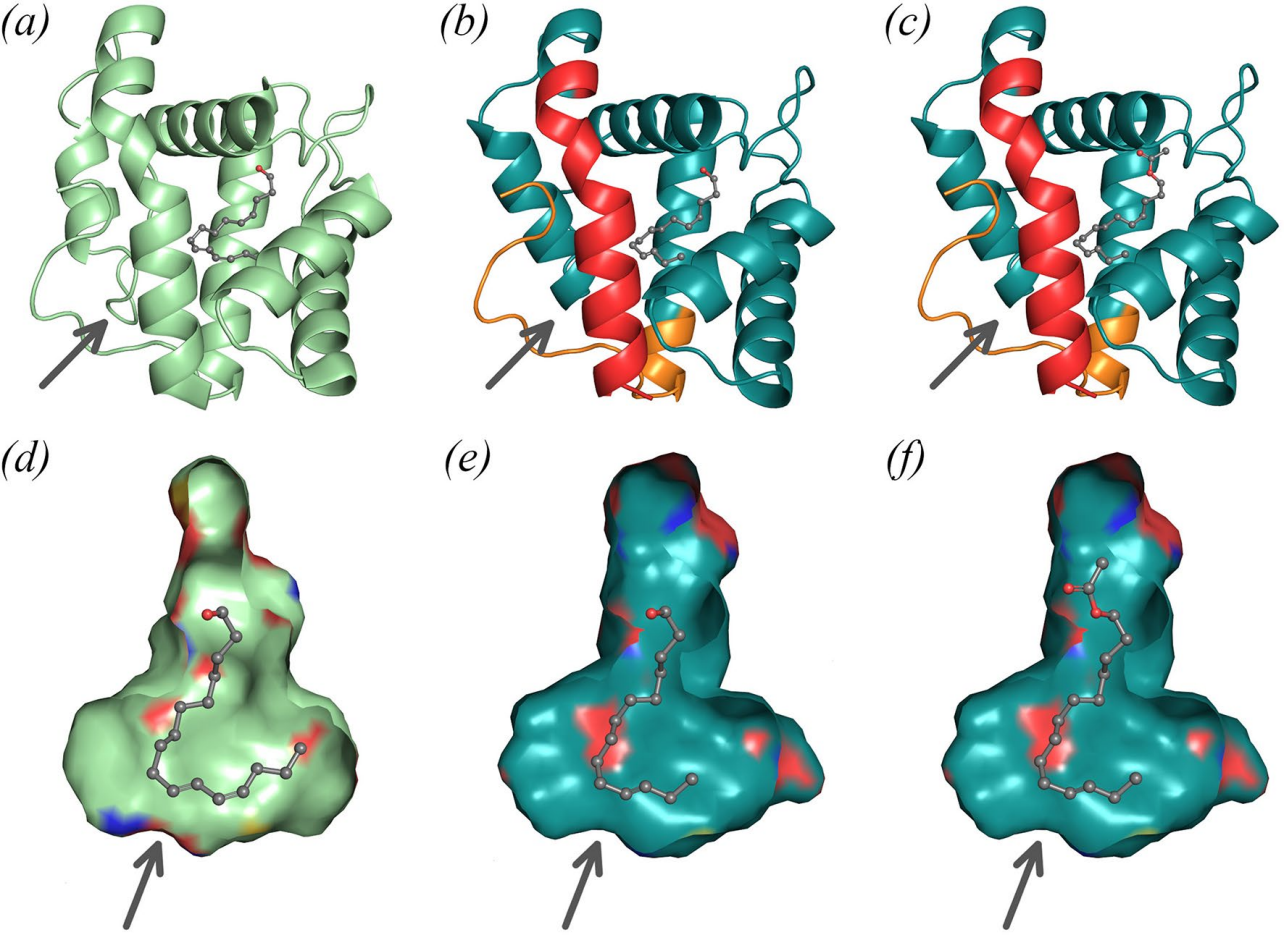
Models for pheromone binding to EposPBP3. (**a**) Structure of BmorPBP1 bound to bombykol, (**b**) hybrid model of EposPBP3 with E11-14:OH and (**c**) hybrid model of EposPBP3 with E11-14:OAc. On (**b**,**c**), the experimentally-determined structure of EposPBP3 is shown in teal while the N- and C-termini built by homology modeling using the BmorPBP1 structure as template are shown in red and orange, respectively. (**d**) Internal cavity of BmorPBP1 with bound bombykol, (**e**) internal cavity of EposPBP3 in closed form with E11-14:OH and (**f**) internal cavity of EposPBP3 in closed form with E11-14:OAc. On (**d**–**f**), the coloured patches on the surfaces indicate the presence of oxygen (red), nitrogen (blue) and sulphur (yellow) atoms pointing towards the surface. Pheromone compounds are shown in sphere mode, with carbon and oxygens atoms coloured in grey and red, respectively. The arrows indicate the approximate location of the cavity opening shown on Fig. 3b. Image drawn using PyMOL Molecular Graphics System, Version 2.0 (https://pymol.org/2/).

Taking advantage of the close sequence and structural similarities between EposPBP3 and two other lepidopteran PBPs that have been co-crystallised with pheromone components (BmorPBP1 bound to bombykol^5^ and AtraPBP1 bound to Z11, Z13-16:OH and to Z11, Z13-16:OAc^13^; BmorBPB1 and AtraPBP1 share 51% and 53% sequence identity with EposPBP3, respectively), we next investigated the binding mode of E11-14:OAc and E11-14:OH to EposPBP3. In both co-crystal structures, the bound pheromones adopt a conserved hook-shaped conformation and bind in enclosed hydrophobic cavities of similar shapes located at the centre of both proteins^5,13^. Since our structure of EposPBP3 adopts a partially open conformation which makes it more difficult to assess ligand binding, we decided to build a “hybrid” model of EposPBP3 in a closed conformation by merging the N- and C-termini (residues 1–19 and 121–137, respectively) of a homology model of EposPBP3 built on BmorPBP1 in its closed state (PDB 1DQE), with the core domain (encompassing residues 20–120) of our experimental structure (Fig. 4b). Structural superimpositions of this “hybrid” closed EposPBP3 structure with BmorPBP1 had r.m.s deviations of 1.51 Å and 1.93 Å for Cα atoms and all atoms, respectively, and showed that the shapes of the internal cavities of these two proteins (and of that of AtraPBP1) are very similar. This is not surprising since 10 of the 22 residues shaping the internal cavities of BmorPBP1 and EposPBP3 are strictly conserved, while the remaining 12 have a high degree of similarity. Altogether, this suggests that the pheromone components of *E. postvittana* bind to EposPBP3 in a conformation similar to that observed for the pheromones bound to BmorPBP1 and to AtraPBP1. Because the experimental electron density map of bombykol in the BmorPBP1 structure is much better resolved than that of the alcohol or aldehyde pheromones in AtraPBP1, we used the conformation of bombykol as a template to model both E11-14:OH and E11-14:OAc in our “hybrid” EposPBP3 closed structure, and subsequent structural comparisons (discussed below) were performed with respect to the BmorPBP1/bombykol structure. As shown in Fig. 4, both hook-shaped compounds fit remarkably well inside the cavity of EposPBP3.

## Discussion

Here we have experimentally determined the three-dimensional structure of a carrier protein that displays male-biased antennal expression in *E. postvittana* and is capable of binding at least two of the known sex pheromone compounds used by this moth. This protein, therefore, likely has a critical role in reproduction in *E. postvittana* in that it is responsible, at least in part, for transporting volatile chemical cues from the environment to the odorant-specific receptors embedded in OSNs within the moth sensilla. The results presented here provide the first structural data for any olfactory proteins from the Tortricid family of moths, one who’s members are responsible for causing tremendous economic damage to horticultural crops worldwide.

In this study, several attempts to express EposPBP3 in *E. coli* resulted in contrasting results. A large amount of recombinant protein was initially expressed and purified using a cleavable N-terminal His-tag, however crystallisation trials with that construct (either with or without the His-tag) were unsuccessful. In contrast, bacterial expression of EposPBP3 with a C-terminal His-tag resulted in low levels of protein being produced despite using a vector and strains optimized for recombinant protein production. In this case, of the recombinant protein that was produced, we found that the majority was trapped within the periplasm and was not being secreted, which left little to no yields after purification from *E. coli* cell lysates. We then attempted to express EposPBP3 in Sf9 cells using DH10Bac which was not successful, although using the MultiBac and EmBacY systems allowed very low levels of expression in these cells. We found that using the EmBacY virus in High Five cells generated relatively high levels of expression and also secreted the protein into the media in a similar manner to that found in vivo. The EmBacY baculovirus^27^ is a derivative of the Multibac bacmid which has an enhanced, integrated yellow fluorescent protein-coding gene (YFP). The parental MultiBac virus has been designed to reduce proteolysis of overexpressed proteins through a deletion of *v-cath*, a viral protease and its activator^28^, which may explain why the MultiBac and EmBacY systems performed better than the DH10Bac system.

Prior to the initiation of attempts to crystallise the protein and experimentally determine its structure, we sought to validate the quality (e.g., proper folding) of the protein through its ability to bind to the pheromone compounds that it is proposed to transport in vivo. One commonly used assay system for measuring PBP/OBP function is to conduct competitive binding experiments using potential ligands and the fluorescent probe, NPN^29,30^. In our functional experiments we were able to observe increased fluorescence upon addition of recombinant EposPBP3 to assay buffer containing NPN, as well as subsequent decreases in fluorescence upon addition of relevant pheromone compounds to wells containing EposPBP3-bound NPN. These results confirmed that our recombinant protein was functional in that it formed a binding pocket through which E11-14:OAc and E11-14:OH were able to compete ‘off’ NPN. However, in our assays we found that one of the pheromone compounds we used, E11-14:OAc, interacted in such a way with NPN that it increased the fluorescent emissions of the solution upon addition. While subsequent reductions in fluorescence upon addition of E11-14:OAc to EposPBP3/NPN were measurable, this phenomenon of ligand interacting directly with NPN posited a limiting factor to the applicable use of this compound in further PBP-pheromone binding studies due to the unknown mechanism and complexity of the ligand-NPN interaction. Surprisingly, to our knowledge, in all of the reports on insect PBP-pheromone binding, only two studies^31,32^ report similar cross-reactivity of odorant or pheromone molecules interacting directly with NPN, despite the widespread use of structurally similar compounds. In these studies, the authors suggest that the pheromone compounds are actually forming reversed micelles that encapsulate the fluorescent reporter and lead to increases in fluorescent spectra. More recently, the accuracy of affinity measurements of binding proteins for their ligands obtained through the use of NPN (as well as 1-aminoanthracene, an alternate fluorescent probe used for competitive binding assays) has been questioned as it was shown that the results can be drastically, and unexplainably, variable, depending on which type of probe is used^33^. Taken together, these findings dissuaded us from pursuing further binding studies with our recombinant protein. For us, our aim in conducting these analyses was simply to show that the protein was functional, for the sole purpose of gaining confidence in the accuracy of the crystal structure that we subsequently obtained.

The structure determination process of EposPBP3 was not as straightforward as expected. Initial attempts to solve the structure by molecular replacement with Phaser^34^ using various insect PBP/OBP structures as starting models failed. When using the automatic procedure implemented in MoRDa, only one successful solution was found (using an initial model derived from the 1DQE structure), while other attempts using closely related structures (e.g., 4INW, *A. transitella* PBP1; 2FJY, A-form of BmorPBP1; 2WC5, BmoriGOBP2; 2GTE, *Drosophila melanogaster* OBP ‘LUSH’; 3Q8I, *Anopheles gambiae* OBP4; the sequence identities of these proteins with Epos PBP3 are 53, 51, 34, 19 and 19%, respectively) were unsuccessful (Supplementary Fig. S2). Ultimately the one solution found by MoRDa turned out to be correct, although initial electron density maps were very noisy, with only secondary structure elements and the disulphide bridges being clearly observed. The quality of the electron density maps improved significantly after density modification and automatic model building in Autobuild, allowing completion of the structure through additional cycles of manual building and refinement. A representative portion of the final electron density map is shown in Supplementary Fig. S3. The difficulties in finding a molecular replacement solution likely result from the arrangement of the two molecules in the asymmetric unit, where both face each other with extended parts of their N- and C-termini being disordered. Hence, the presence of either an ordered N-terminal α-helix or an elongated C-terminal coil (or both) observed in OBP/PBP structures used as molecular replacement starting models creates packing issues for the molecular replacement. That being said, the model used by MoRDa for the successful run has a folded N-terminal helix (that was later removed during model building and refinement) and it therefore remains unclear why this model lead to a successful molecular replacement solution when other closely related ones did not. This was also observed a posteriori in Phaser, when further attempts to solve the structure using the MoRDa monomer model as a starting model were readily successful (Top LLG = 855 and Top TFZ = 22.7) despite some sidechains clashing at the protomer/protomer interface while other structurally related models remained unsuccessful.

Among the final statistics listed in Table 3, the overall B-factor appears high (90.8 Å^2^) for a 2.60 Å resolution structure. The high resolution cut-off for the data was chosen using the paired-refinement concept introduced in 2012^35^. As described in Table 2, the CC(1/2) value for the high resolution shell (2.72–2.60 Å) is 0.644 (i.e*.*, above the threshold value of 0.5 for this indicator). Subsequently, the 2.60 Å high resolution limit was chosen as a compromise between the CC(1/2) ≥ 0.5 criteria and 〈*I*/σ(*I*)〉 ≥ 1.5, noting that the resolution limit at which 〈*I*/σ(*I*)〉 ≥ 2.0 was 2.73 Å (Table 2). Table 2 also reports the experimental Wilson B-factor (72.7 Å^2^) for the data estimated by Truncate, which was confirmed by alternative data analysis software (B_Wilson_ = 70.2 and 82.6 Å^2^ using Xtriage or SF Check, respectively). This large Wilson B-factor is in agreement with our observations of high disorder within the crystal, where 41 out of 159 residues of each protomer were not observed in electron density maps. It is also consistent with the large average B-factor reported for the refined model. A search in the Protein Data Bank showed that among the 1608 structures of similar resolution (2.50 – 2.70  Å) with similar Wilson B-factors (60–90 Å^2^), 247 (15%) have average B-factors ≥ 85 Å^2^, similar to ours. In our case, the compounding effects between the high disorder in the crystal and the fact that, because of the unusually long incubation time required to obtain the crystals, neither crystal growth conditions nor harvesting or cryopreservation conditions could be optimized, provide a rationale for the high average B-factor reported for the structure presented here.

Of the two PEG molecules modelled at the protein interface, one has clear electron density in both omit and polder omit maps, while the maps are noisier for the second PEG molecule (Supplementary Fig. S1). Because both sit close to the N- and C-termini of the two protein molecules, the possibility that these stretches of electron density result from some partially disordered extended polypeptide chains rather than PEG was investigated. However, attempts to model additional residues (either from the N- or the C-terminal end of the molecule) in place of PEG were unsuccessful. Due to the very long incubation time required to obtain the crystals, we cannot ascertain whether the lack of interpretable electron density at the N- and C-termini of both molecules results from static disorder or from partial proteolysis that may have occurred during crystallisation. We also cannot elaborate on the pH in which crystals were ultimately obtained since it may have significantly shifted from its original value (pH 6.5) in the reservoir solution used. Despite these uncertainties, this new PBP structure is in agreement with the general fold of PBPs/OBPs and is consistent with the large structural plasticity observed for lepidopteran PBPs/OBPs to bind and transport their ligands.

It is noteworthy that within the internal cavities of EposPBP3 and BmorPBP1, four amino acid differences (Leu61Met, Leu76Phe, Val91Ile, Leu94Val) cluster near the end of the bound pheromone. However, these do not trigger any particular steric constraints in EposPBP3 and therefore do not provide any rationale for increased selectivity of EposPBP3 towards shorter C14 pheromones, compared to the C16 pheromone of BmorPBP1. This is in line with previous findings that suggest PBPs may not have great selectivity towards the ligands they bind to but rather play a role in both solubilizing and protecting pheromones during their journey through the sensillum lymph^2^. That being said, a distinctive difference between EposPBP3 and BmorPBP1 is the ability of EposPBP3 to bind both the ester (acetate) and the alcohol derivatives of the E11-C14 compounds, while BmorPBP1 was shown to bind bombykol and its aldehyde derivative, bombykal^36,37^. Although antennae of *B. mori* moths were shown to respond to (*E,Z*)-10,12-hexadecadienyl acetate (bombykyl acetate), in addition to bombykol and bombykal^38^, there has been no direct evidence yet that bombykyl acetate also binds to BmorPBP1. In the structure of the complex between BmorPBP1 and bombykol, the alcohol group of bombykol forms a hydrogen bond with Ser56, which is conserved in EposPBP3 and other lepidopteran PBPs. However, the top part of the cavity (which surrounds and extends above the functional group of the pheromone) appears significantly wider in EposPBP3 than in BmorPBP1 (Fig. 4d,e). This results from the combination of four amino acid substitutions between the two proteins (Val53Met, Ile62Leu, Lys110Trp and Ala111Thr), and from a slight variation in the position of the exposed loop located between helix 3 and helix 4. Such observations are in agreement with the ability of EposPBP3 to not only bind the alcohol but also the bulkier ester pheromone (Fig. 4f). Conversely, since Trp110 and Met53 are distinctive features of BmorPBP1 over other lepidopteran PBPs and GOBPs^5^, this raises the possibility that these residues may play a role in restricting binding of bulkier acetate compounds to BmorPBP1.

Pheromone binding proteins are often lauded as targets for the development of novel pest control technologies, because interfering with or inhibiting carrier proteins, such as EposPBP3, could theoretically prevent the chemical cues emitted by female conspecifics—the sex pheromones—from being converted to action potentials that stimulate behavioural responses in the male insect. While there is thought to be some redundancy in function in insect OBPs and PBPs, in that they are broadly tuned with overlapping binding specificities, the opportunity exists to target these proteins as an avenue to influence population dynamics in horticultural pests. Perturbing the ability of PBPs to efficiently transport pheromone compounds to their receptors would function similarly to the technique of mating disruption, in which reproductive cycles, and therefore population growth, are affected by minute delays in a pest’s ability to detect and process sex pheromone signals through the desensitization of OSNs housing sex pheromone receptors. One can imagine using the structural data presented here to conduct structure–activity-relationship studies through which synthetic molecules are developed to outcompete natural sex pheromones for binding sites on EposPBP3. However, as previously mentioned, such strategies carry inherent risks if they do not target the pest insect specifically^15^. Ultimately, modern advances in the development of molecular-based pest control technologies will never serve as a magic bullet, capable of completely controlling horticultural pests. Instead, any advances along these axes will simply augment the currently available control tactics of integrated pest management (IPM). The next wave of advancements in the molecular approach to controlling agricultural pests, and therefore development of tools to augment IPM, will likely be based on structural data like those presented here in which proteins with critical biological functions are experimentally characterized.

## Methods

### Recombinant protein production

For expression of the N-terminal His-tagged EposPBPB3 construct in bacteria, the sequence was identified from an *E. postvittana* EST dataset^24^. Primers were designed to the 3′ and 5′ ends and PCR products were cloned into pGEM-T Easy (Promega) and sequenced. Correct sequences were ligated into a pET30a vector (Novagen) to produce an N-terminally His-tagged protein. The protein was expressed in Rosetta Gami 2 cells and purified in four steps (affinity chromatography, anion exchange, tag removal followed by affinity chromatography and gel filtration). For the C-terminal His-tag constructs (Table 1), the full-length ORF of EposPBP3 was designed from the transcriptome^25^ with a C-terminal 10-Histidine tag separated by a TEV cleavage site and was manufactured by Geneart (ThermoFisher). EposPBP3 was cloned into the optimized bacterial expression vector, pET301 (Thermo Fisher Scientific), and expressed in *E. coli* strains BL21 and Rosetta. Insufficient quantities of recombinant protein were produced using this approach, and recombinant production methods were then switched to the baculoviral-Sf9 system. The C-terminally tagged EposPBP3 construct was gateway cloned into the pDEST8 vector and transformed into DH10Bac cells as per the manufacturer’s protocol. Bacmids were purified according to the Bac-to-Bac manual and transfected into Sf9 cells to generate high-titre virus stocks. Hi5 cells at 1 × 10^6^ cells/mL were infected with virus at an MOI of 1 and incubated at 27 °C with shaking at 120 RPM for 72 h. The cells were centrifuged at 8000*g* for 20 min, and the supernatant was passed through a 0.22 µm filter prior to being applied to a 5 mL NiNTA Excel column (Qiagen). The column was washed with 10 column volumes of 20 mM Tris/HCl pH 7.5, 300 mM NaCl and 20 mM imidazole followed by 10 column volumes of 20 mM Tris/HCl pH 7.5, 100 mM NaCl and 50 mM imidazole, and eluted with 5 column volumes of 20 mM Tris/HCl pH 7.5, 100 mM NaCl, 500 mM imidazole. Despite the presence of the TEV cleavage site within the construct, removal of the C-terminal 10-Histidine tag was not performed and the uncleaved protein was further purified by size exclusion chromatography in Tris/HCl pH 7.5, 100 mM NaCl. Peak fractions were collected and analysed by SDS-PAGE and western blot. The protein was concentrated to 16.8 mg/mL and frozen at − 80 °C until use in either functional studies or crystallisation screens.

**Table 1:** Recombinant protein production information.

Source organism	*Epiphyas postvittana*
DNA source	Synthetic
Cloning vector	pDEST8
Expression vector	Baculovirus (bMON14272 and pMON7124)
Expression host	*Spodoptera frugiperda* (*Sf9*)
Complete amino acid sequence of the construct produced	MARLSILVALVVLGVNISEIDSSEEVMKDLTSGF IKVLEECKKELNLSESIINDLYNYWKEDYSLLNR DVGCAIVCMSKKLELIDTSGKIHHGNAEDLAKK HGADSEVAAKLVAILHECEKTHDAIEDQCMKAL EIAKCFRTNIHELNWAPKMDVVITEVLTEVENLY FQGHHHHHHHHHH^a^

^a ^The underlined sequence corresponds to the signal peptide that is predicted to be cleaved off upon secretion of the protein from the cells.

### Functional testing of recombinant protein

Recombinant EposPBP3 was tested for functionality, and therefore proper folding, through its ability to bind to E11-14:OAc and E11-14:OH in a competitive binding assay using the fluorescent indicator N-phenyl-1-napthylamine (NPN, Sigma Aldrich) and a SpectraMax Platereader (Molecular Devices). Pheromone stocks were purchased from Beduokian Research and were all at least 96% pure, the control compound, tetradecane, was purchased from Sigma-Aldrich. All fluorescence binding assays were conducted using Tris-HCl buffer (pH 7.4) containing 2% ethanol, black-walled, clear-bottom, 96-well plates, and an excitation wavelength of 337 nm and emission spectra of 370–470 nm. First, intrinsic EposPBP3 fluorescence was determined at 2 μM in buffer solution, then NPN was added to the 2 μM EposPBP3 solution at various concentrations (2, 4, 6, 8 and 10 μM) to determine optimal, intrinsic fluorescence-blocking concentrations. Changes in NPN fluorescence were then measured after adding various concentrations of EposPBP3 to solutions containing 10 μM NPN. Finally, competitive binding assays were conducted by adding E11-14:OAc, E11-14:OH or tetradecane (negative control) to wells (10 μM, final concentration) containing 10 μM NPN and various concentrations of EposPBP3. Changes in fluorescence were calculated as the mean change in maximum fluorescence following the addition of test compounds from three experiments using fresh stocks of protein, NPN and test compounds.

### Crystallisation

Crystallisation experiments were performed by hanging drop vapour diffusion at 20 °C. 200 μL sparse-matrix screens from Molecular Dimensions (Structure Screen 1 + 2 and JCSG-plus, MIDAS-plus and MORPHEUS) were used as reservoir solutions in 96-well flat-bottom Greiner plates. Drops consisting of 1 μL EposPBP3 16.8 mg/mL and 1 μL reservoir solution were dispensed manually on a qPCR film (Axygen UC-500), which was then turned over and sealed over the reservoirs. Drops were checked regularly over a 6-month period without any crystals observed. At a final inspection after ~ 11 months, however, crystals were observed in two conditions of the Structure Screen, SS2-22 and SS2-46.

### Data collection and processing

The crystals were cryoprotected by successive soaking into their respective reservoir solutions supplemented with 15, 25 and 35% glycerol. Datasets were collected at the Australian Synchrotron MX2 beamline. Datasets were indexed and integrated with XDS^39^ and scaled/merged with Aimless^40^ from the CCP4 suite^41^. The statistics for data collection and processing are listed in Table 2.

**Table 2:** Crystallographic data collection and processing information. ^a^〈I/σ(I)〉 = 2.00 at 2.73 Å resolution. The 1.5 cut-off value at 2.60 Å resolution was chosen based on the CC(1/2) value above 0.5 observed in the outer resolution shell.

Diffraction source	Australian synchrotron; MX2
Wavelength (Å)	0.95372
Temperature (K)	100
Detector	Eiger X 16M
Crystal-detector distance (mm)	300
Rotation range per image (°)	0.1
Total rotation range (°)	360
Exposure time per image (s)	0.01
Space group	*P*4_3_
*a*, *b*, *c* (Å)	53.38, 53.38, 105.95
α, β, γ (°)	90, 90, 90
Mosaicity (°)	0.12
Resolution range (Å)	47.670–2.600 (2.720–2.600)
Total no. of reflections	130,814 (16,455)
No. of unique reflections	9187 (1116)
Completeness (%)	100.000 (100.000)
Redundancy	14.200 (14.700)
〈*I*/σ(*I*)〉	12.400 (1.5^a^)
*CC*(1/2)	0.999 (0.644)
*R*_r.i.m_	0.117 (2.329)
*R*_p.i.m_	0.031
Overall *B*-factor from Wilson plot (Å^2^)	72.7

### Structure solution and refinement

The structure was solved by molecular replacement with MoRDa^42^ on the CCP4-online server, using the structure of BmorPBP1^5^ as a model (PDB entry 1DQE) and searching for two molecules in the asymmetric unit (MoRDa Q score = 0.649). At this stage, secondary structure elements and the three disulphide bridges were clearly observed in the electron density map, but the overall quality of the map was quite poor. Density modification and automatic model building were then performed with AutoBuild in Phenix^43^, which built 217 out of 318 residues into much improved electron density maps. Subsequent cycles of model building and refinement were performed in Coot and Refmac5, respectively^44,45^. Two 2-(2-Methoxyethoxy) ethanol moieties (PEG fragments) were modelled at the dimer interface in the late stages of the refinement. The final model was refined using the optimized parameters obtained from the PDB_redo server^46^. The refinement statistics are listed in Table 3.

**Table 3:** Structure refinement statistics.

Resolution range (Å)	47.6600–2.6000 (2.6670–2.6000)
Completeness (%)	100.0
σ cutoff	*F* > 0.000σ(*F*)
No. of reflections, working set	8653 (651)
No. of reflections, test set	505 (34)
Final *R*_cryst_	0.216 (0.335)
Final *R*_free_	0.259 (0.391)
Cruickshank DPI	0.7092
No. of non-H atoms	1860
Protein	1835
Ligand	16
Solvent	9
Total	1860
**R.m.s. deviations**	
Bonds (Å)	0.007
Angles (°)	1.377
**Average B-factors (Å**^**2**^**)**	
Protein	90.8
Ligand	119.9
**Ramachandran plot**	
Most favoured (%)	96
Allowed (%)	4

### Molecular modelling

A homology model of EposPBP3 was built in Modeller^47^ using the BmorPBP1 structure (PDB 1DQE) as a template. The hybrid model for EposPBP3 in the closed form was then obtained by manually merging the N- and C-termini (residues 1–19 and 121–137, respectively) of the homology model with the core domain (encompassing residues 20–120) of our experimental structure. 3D coordinate files for E11-14:OH and E11-14:OAc were obtained from PubChem, and restraints description files (cif files) were generated in JLigand from the CCP4 suite. The coordinates and structure factors of the BmorPBP1/bombykol structure (PDB 1DQE) were loaded in Coot. E11-14:OH and E11-14:OAc were then fit inside the experimental electron density map of bombykol, followed by minor manual adjustments guided by the conformation of bombykol in the structure.

## Supplementary information

Supplementary Information.

## Data Availability

Structure factors and coordinates have been deposited to the Protein Data Bank, with entry code 6VQ5.
