# Supplementary material for: Crystal structure of Epiphyas postvittana pheromone binding protein 3
Source: Sci Rep. 2020 Oct 1;10:16366. doi: 10.1038/s41598-020-73294-8 (PMC7530677; doi:10.1038/s41598-020-73294-8)

Crystal structure of *Epiphyas postvittana* pheromone binding protein 3

**Cyril Hamiaux, Colm Carraher, Christer Löfstedt, Jacob A. Corcoran**

**Supplementary Material**

**Figure S1.** Final sigmaA-weighted 2m*Fo*-D*Fc* maps contoured at 1.3σ (*a, b*), omit m*Fo*-D*Fc* maps contoured at 3.0σ (*c, d*) and polder omit maps m*Fo*-D*Fc* contoured at 3.0σ (*e, f*) around the 2 PEG molecules. Images drawn using Coot software, Version 0.8.9.2 ([https://www2.mrc-lmb.cam.ac.uk/personal/pemsley/coot/](https://gcc02.safelinks.protection.outlook.com/?url=https%3A%2F%2Fwww2.mrc-lmb.cam.ac.uk%2Fpersonal%2Fpemsley%2Fcoot%2F&data=02%7C01%7C%7C244d8e2bf85d432f912b08d83274f8cb%7Ced5b36e701ee4ebc867ee03cfa0d4697%7C0%7C0%7C637314825269824583&sdata=pNskJykTMBp4617SfhE9DN9xh0pHHQ8Mo1YLlGhqmxY%3D&reserved=0)).


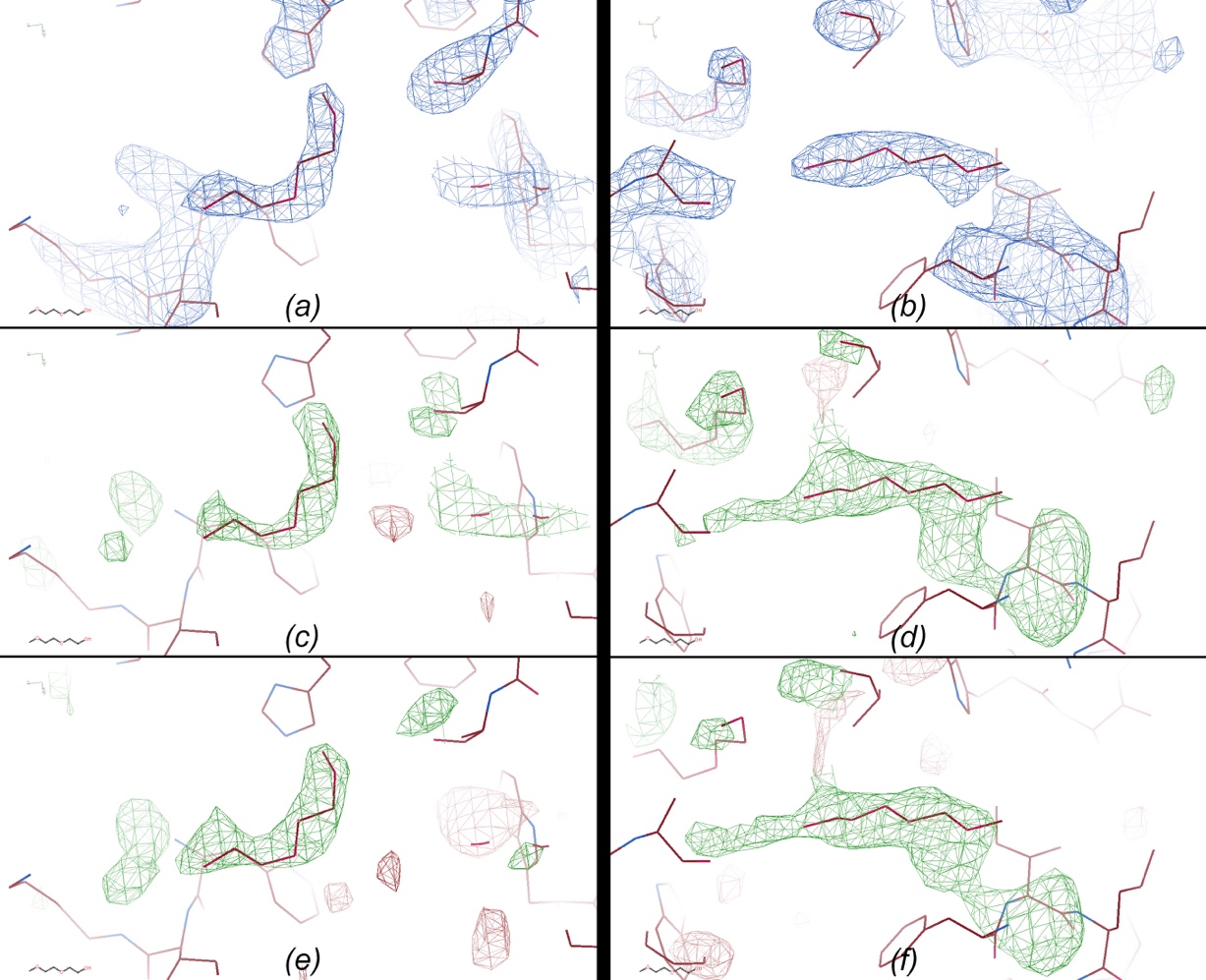


**Figure S2.** MoRDa output summary.


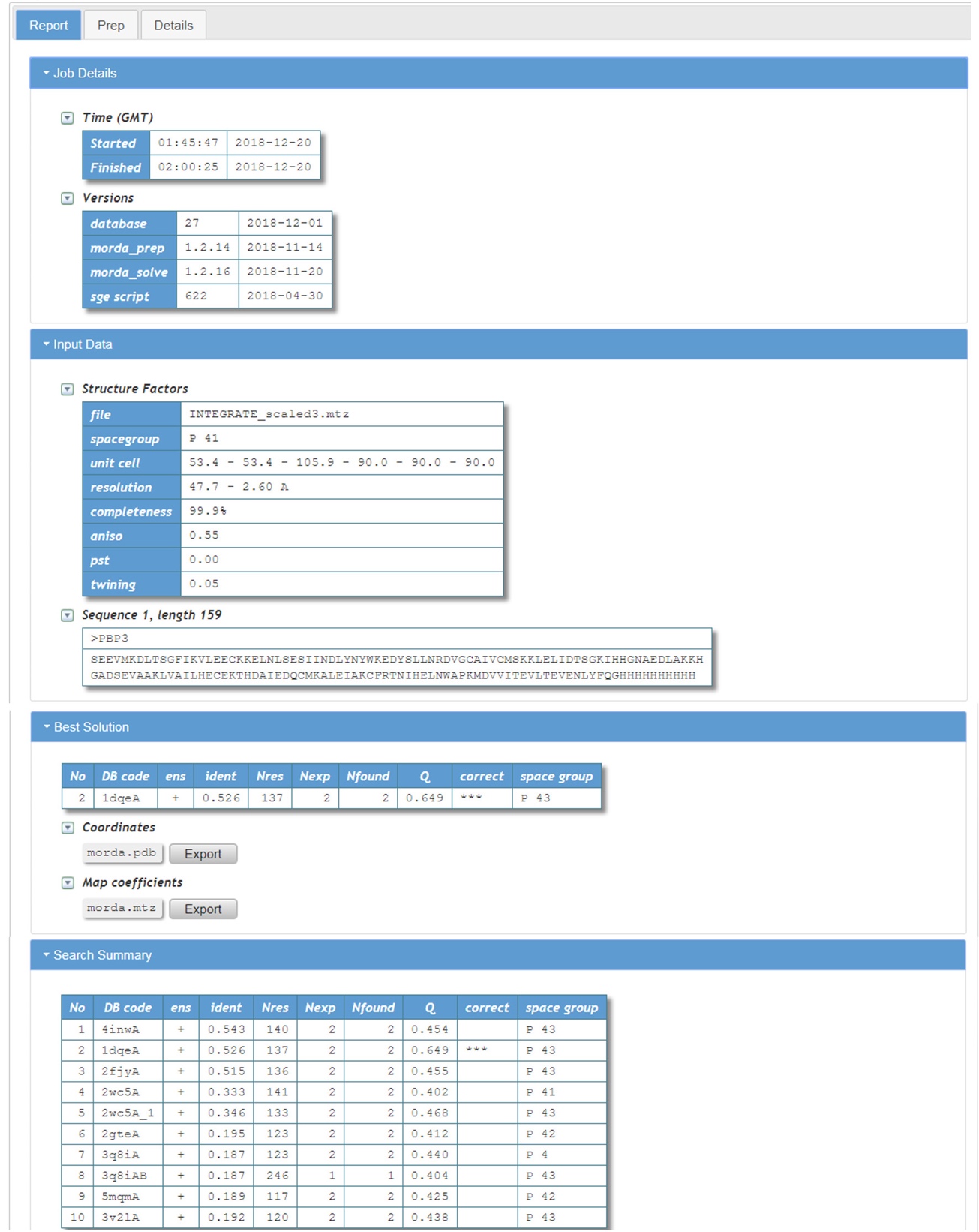


**Figure S3.** Stereo image of a portion of the electron density maps of EposPBP3. The Final sigmaA-weighted 2mFo-DFc map contoured at 1.0 σ is shown in blue. The difference mFo-DFc map contoured at 3σ is shown in green. EposPBP3 is shown in stick mode with carbon atoms in green, oxygen atoms in red, nitrogen atoms in blue and sulphur atoms in yellow. The disulphide bridge shown links Cys97 and Cys117. Images drawn using Coot software, Version 0.8.9.2 ([https://www2.mrc-lmb.cam.ac.uk/personal/pemsley/coot/](https://gcc02.safelinks.protection.outlook.com/?url=https%3A%2F%2Fwww2.mrc-lmb.cam.ac.uk%2Fpersonal%2Fpemsley%2Fcoot%2F&data=02%7C01%7C%7C244d8e2bf85d432f912b08d83274f8cb%7Ced5b36e701ee4ebc867ee03cfa0d4697%7C0%7C0%7C637314825269824583&sdata=pNskJykTMBp4617SfhE9DN9xh0pHHQ8Mo1YLlGhqmxY%3D&reserved=0)).


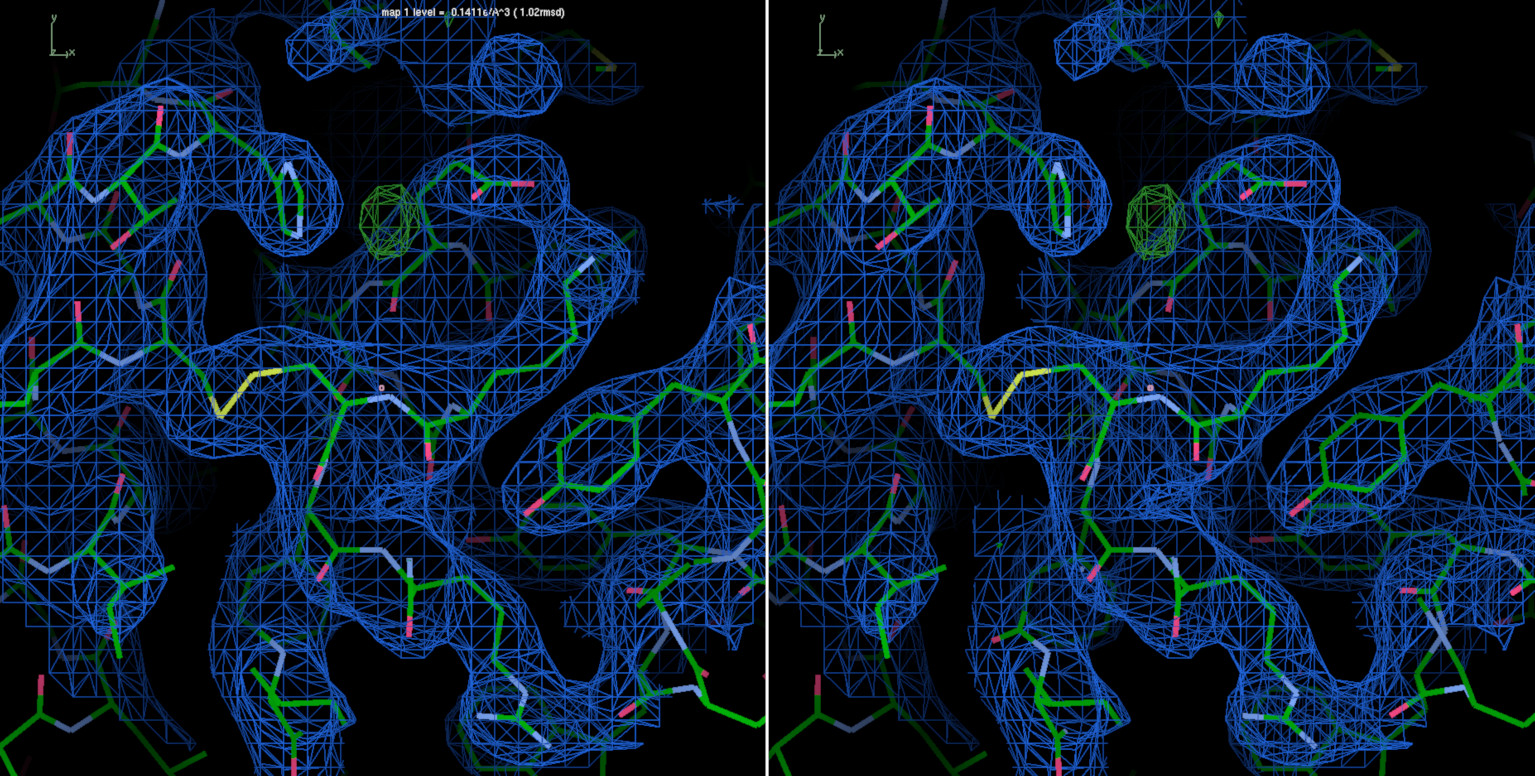

Supplement: Supplementary file 1 — Supplementary Information. [file 41598_2020_73294_MOESM1_ESM.docx]
